# Association between Stress and Social Support Perceived among Undergraduate Health Sciences Student

**DOI:** 10.21315/mjms2023.30.3.16

**Published:** 2023-06-27

**Authors:** Nurul Azizah Abdul Aziz, Nur Sakinah Baharudin, Noor Amiera Alias

**Affiliations:** 1 Faculty of Health Sciences, Universiti Teknologi MARA, Selangor, Malaysia; 2 Faculty of Health Sciences, Universiti Teknologi MARA, Pulau Pinang, Malaysia

**Keywords:** COVID-19, undergraduate, stress, social support

## Abstract

**Introduction:**

A high level of perceived social support can lessen stress. However, the lack of knowledge on stress and perceived social support among students during the coronavirus disease (COVID-19) pandemic was explored. Thus, this study aimed to investigate the relationship between stress and perceived social support among undergraduate Health Sciences students.

**Methods:**

A convenience sampling method was used in a cross-sectional study of 290 undergraduate Health Sciences students in public universities. The Perceived Stress Scale (PSS-10) was used to measure the perception of stress, and the Multidimensional Scale of Perceived Social Support (MSPSS) was used to measure perceived social support from three sources, including family, friends and significant others.

**Results:**

A statistically significant correlation was found between the stress level and the total score of the MSPSS (*r* = −0.432), perceived social support from family (*r* = −0.429), significant others (*r* = −0.328), and friends (*r* = −0.219, *P* < 0.001). Over three-quarters (73.4%) of the students have a moderate stress level (mean = 21.17, SD = 5.75). The highest social support perceived was from a family (mean = 5.21, SD = 1.48).

**Conclusion:**

The study suggested that social support from family is the strongest for students to go through the stress of tough times. It also highlighted the need for attention to stress management among undergraduate students for healthy well-being. Future studies that involve other academic fields of study and qualitative research would give useful information on perceived social support among students.

## Introduction

The world was shocked by the highly pathogenic coronavirus disease 2019 (COVID-19), which primarily spread in mid-December in Wuhan, Hubei Province, China (1). On 30 January 2020, the World Health Organization (WHO) announced severe acute respiratory syndrome COVID-19 as a Public Health Emergency of International Concern. This crisis adversely impacted every society worldwide, contributing to the psychological pressures on people, including students.

In Malaysia, the Movement Control Order (MCO) was enforced as a precaution to avoid widespread transmission among the public at the early stage of the pandemic. With the MCO, the educational institutions were instantly changed to remote online classes, and the Malaysian population was confined and socially isolated for a long period. Social distancing, emergency remote teachings, and uncertainty and delays in the initiation of schools, colleges and universities were implemented, which significantly impacted the psychological well-being of the students (2). The entire educational system was in a challenging situation as most undergraduate students had to continue their studies online following the government requirement.

A recent study stated that negative social consequences and the COVID-19 pandemic contributed to adverse psychological outcomes, such as anxiety, stress and depression which indirectly impacted daily activities (3). Lazarus and Folkman (4) defined stress as ‘a specific relationship between the person and the environment that is appraised by the person as taxing or exceeding his/her resources and endangering his/her well-being’. A review of the literature on the prevalence of stress, anxiety and depression among the general population during the COVID-19 pandemic has identified that COVID-19 not only causes physical health concerns but also leads to several psychological disorders that can impact the mental health of people in different communities (5). With the growing number of infected people, it is necessary to address mental health concerns, as the psychological consequences of COVID-19 could be serious (1).

The study conducted among undergraduate Health Sciences students in Sri Lanka showed that most students experienced a high level of stress (82.6%) (6). In Malaysia, more than half of the medical students (56%) are stressed, indicating that stress among the students is worrying (7). Besides, a high prevalence rate of perceived stress among undergraduate students in Malaysia showed that undergraduate students are at risk for stress-related disorders (8).

Moreover, social support has been recognised as one factor in improving emotional balance. Cohen and Wills (9) defined social support as a process through which social relationships promote health and well-being. Generally, social support is one of the essential variables which can promote resilience to stress (10). Other evidence indicate that social support was a protective factor for mental health, especially during the outbreak of COVID-19 (11). Thus, improving social connection and support should be highlighted to cope with the adverse psychological consequences of the COVID-19 pandemic (12). According to research, social support has a significant negative association with psychological issues such as stress highlighting the importance of social support in students’ well-being (13–15).

The requirement to utilise more resources to understand how social support impacts individuals to cope with their psychological issues should be highlighted. The fact that COVID-19 is perceived as a challenging situation for students has been a concerning topic among researchers recently. However, more research on stress and social support among university students is still needed, especially during the COVID-19 pandemic. It is crucial to assess the perceived stress of Health Sciences students to ensure sufficient support for the vulnerable group during the pandemic. Therefore, in this study, the significant association of social support as an essential aspect for individuals who encountered stress was further examined with the following objectives:

- To identify the stress level among undergraduate Health Sciences students
- To determine perceived social support by undergraduate Health Sciences students
- To determine the correlation between the perceived social support and stress levels among undergraduate Health Sciences students

## Methods

### Participant

The present study is a cross-sectional study conducted among 290 undergraduate Health Sciences students from five Malaysian public universities (Universiti Teknologi MARA [UiTM], Universiti Sains Malaysia [USM], Universiti Kebangsaan Malaysia [UKM], Universiti Sultan Zainal Abidin [UniSZa] and Universiti Putra Malaysia [UPM]). The participants consisted of full-time university students from the Faculty of Health Sciences, able to understand and read the English language. The age of participants was between 19 years old and 28 years old. Part-time university students, postgraduate students and non-Health Sciences students were excluded from the study.

### Instruments

#### Perceived Stress Scale (PSS-10)

The PSS-10 (16) is a short survey assessing an individual’s stress perception. The PSS-10 consists of 10 questions that are answered on a 5-point Likert scale (0 = never, 1 = almost never, 2 = sometimes, 3 = fairly sometimes and 4 = always). Individual PSS-10 scores can range from 0 to 40, with higher values suggesting greater stress perception. Scores ranging from 0 to 13 would be considered low stress, 14 to 26 would be moderate stress and 27 to 40 would be high perceived stress (16). The total score was obtained by reverse scores for questions 4, 5, 7 and 8, and by summing all item scores. It has been shown that Cronbach’s alpha ranged from 0.84 to 0.86, indicating that the reliability is good for this measure. The PSS has been empirically validated, especially with populations of college students and workers.

#### Multidimensional Scale of Perceived Social Support

This study used the Multidimensional Scale of Perceived Social Support (MSPSS) to measure an individual’s perception of support from three sources, including significant others (special person/counselor), family and friends (17). It is a self-reported assessment instrument that is measured with a 7-point scale (0 = very strongly disagree to 7 = very strongly agree) consisting of 12 items. The MSPSS was divided into three factors which are significant others (scale = 1, 2, 5, 10), family (scale = 3, 4, 8, 11), and friends (scale = 6, 7, 9, 12). The respondents were asked to rate each statement using a 7-point Likert scale ranging from 1 = very strongly disagree to 7 = very strongly agree. The final score was calculated by adding all the items together, and it ranged from 12 to 84. The higher the scores, the higher the perceptions of social support. It has been shown that the internal consistency is between 0.80 and 0.95.

### Sample Size

The sample size in this study was calculated by the Raosoft sample size calculator online software. According to the statistics, the number of students enrolled in the health and welfare field of study in public universities is 35,037 (18). For a 95% level of confidence and 5% margin error, the sample size would be about 380 participants.

### Data Collection Procedures

The data collection commenced from January 2021 until April 2021. A snowball sampling technique was employed in this study. The students from the listed universities from the sample set (UiTM, USM, UKM, UniSZa and UPM) were reached by the researcher through WhatsApp medium. To get the same proportion of numbers for each group strata, samples are taken for each group to meet a quota. The representative from the university agreed to forward the Google Form link to reach more Health Sciences students. The link consisted of an online consent form and the inclusion criteria to participate in this study. The eligible participants were required to fill up the online survey questionnaire consisting of three sections (Sections A, B and C). Section A gathered demographic data on participants’ age, gender and year of study. Section B is the PSS-10 which aimed to assess stress and Section C is the MSPSS which aimed to measure the perception of support. All 290 participants answered the questionnaires and all data were included for analysis.

### Data Entry and Analysis

The Statistical Package for the Social Sciences (SPSS) version 25.0 software was used to analyse the obtained data. A descriptive statistic summarised analysis of the demographic data obtained from the participants. The stress level among the participants was analysed and presented in frequency and percentage, while perceived social support was analysed and presented in means and standard deviation. A correlation analysis, the Spearman correlation coefficient test, was conducted due to non-normally distributed continuous data to determine the relationship between perceived social support and the stress level. A *P*-value of less than 0.05 is considered statistically significant with a 95% confidence interval (CI).

### Ethical Issues

Ethical approval was obtained from the Research Ethics Committee (REC) of UiTM Shah Alam. The participation involved is entirely voluntary. Participants were also informed that their personal information would be kept confidential and utilised strictly for research purposes.

## Results

### Descriptive Statistics of the Demographic Data Profile

The respondent consists of 290 undergraduate Health Sciences students in public universities. Out of 290 students, 11.4% of the participants were males (*n* = 33) and 88.6% were females (*n* = 257). The participants were between the ages of 19 years old and 28 years old. The majority were in the age group 22 years old– 24 years old (65.2%), followed by the age group 19 years old–21 years old (20%) and 25 years old–28 years old (14.8%). Regarding their year of study, the majority were in the Fourth-year (45.5%), followed by the Third-year (29.0%), Second-year (16.9%) and First-year (8.6%) (Table 1).

### Perceived Stress among Undergraduate Health Sciences Students

Table 2 shows the level of stress among undergraduate Health Sciences students. The score interpretation was sub-grouped into three categories. Moderate stress, with a score of 14–26, showed the highest percentage (73.4%), followed by high stress, with a score of 27–40 (17.6%) and low stress, with a score of 0–13 (9%). The mean score on the PSS was 21.17 (SD = 5.75), as given in Table 3.

### Social Support Perceived among Undergraduate Health Sciences Students

The MSPSS was used to explore the participants’ perceived social support. The scale was categorised by the source of perceived social support: i) support from family; ii) support from friends and iii) significant others, which is the availability of a special person. The mean score on the MSPSS was 5.23 (SD = 1.19), as detailed in Table 3. The results showed that the friends’ support subscale (mean = 5.34, SD = 1.33) was the highest mean among all perceived social support subscale scores, while family support subscales (mean = 5.21, SD = 1.48) was the second lowest mean of perceived social support. The significant other support subscale was the lowest mean (mean = 5.15, SD = 1.65). All the social support perceived by the undergraduate Health Sciences students is considered high social support as the range is between 5.1 and 7.0.

### The Relationship between Social Support and Level of Stress among Undergraduate Health Sciences Students

The results of Spearman’s correlation between social support perceived and the stress level among undergraduate Health Sciences students are shown in Table 4. The analysis shows a statistically significant association between the stress level and social support perceived. Based on the result, there was a moderate negative correlation between the stress level with the total social support scale and family scale (*r* = −0.432, *P* < 0.001; *r* = −0.429, *P* < 0.001, respectively), while the negative low correlation with significant other scale and friend scale (*r* = −0.328, *P* < 0.001; *r* = −0.219, *P* < 0.001, respectively). This suggests that students with a high stress level perceive a low level of social support from significant others, family members and friends. Furthermore, students with a high level of social support reported being less stressed.

The result of the correlational analyses presented in Table 5 shows that all correlations were statistically significant with a moderate negative correlation (UiTM; *r* = −0.412, *P* < 0.001; UKM; *r* = −0.411, *P* < 0.001, UniSZa; *r* = −0.409, *P* < 0.001 and USM; *r* = −0.401, *P* < 0.001, respectively), while negative low correlation (UPM; *r* = −0.370, *P* < 0.001, respectively). According to the results of the correlational analyses shown in Table 5, all correlations were statistically significant, with some showing a moderately negative correlation (UiTM; *r* = −0.412, *P* = 0.001; UKM; *r* = −0.411, *P* = 0.001; UniSZa; *r* = −0.409, *P* = 0.001 and USM; *r* = −0.401, *P* = 0.001, respectively), while UPM (*r* = −0.370, *P* < 0.001) had a low negative correlation. In summary, students from any university with a high stress level perceive a low level of social support adversely.

## Discussion

By conducting an online survey during the pandemic, we found that the undergraduate Health Sciences students in this study are mostly at a moderate stress level. The result was similar to some studies among university students during the COVID-19 pandemic, which reported moderate to high levels of perceived stress (19–22). Generally, Health Sciences students had a high prevalence of perceived stress compared to others (23, 24). During the COVID-19 pandemic, students were required to adjust to the new norm and new educational systems as they transitioned from traditional face-to-face learning to an online learning method expected to further exacerbate academic stressors for students (25). This showed that the sudden shutdown of educational institutions adversely impacted the level of stress among students.

A study by Malik and Javed (26) showed moderate to high perceived stress by 96.9% of the respondents, which proved that COVID-19-induced online learning had a negative impact on the psychological health of university students. The social isolation, physical isolation, and a lack of interaction and emotional support were expected to further worsen the psychological state of the students (27). Besides, pressure due to many online tasks, limited resources relevant to the subject, limited and weak internet connection, and the unfamiliar study environment could influence the psychological state of students (28). The difference in stress levels between persons in similar conditions is determined by their stress-coping abilities, with low-stress levels indicating a positive response and the ability to cope well in challenging situations (21). Further research is required to determine the causes of the perceived stress.

According to the findings, social support can significantly impact how undergraduate Health Sciences students perceive stress. Family and friends were found to be the two most common sources of support among undergraduates (44.1% and 33.8%, respectively) in this study, with significant others ranking last. According to Minhat and Alawad (29), a good family relationship is crucial as a coping mechanism for psychological issues. Furthermore, social support from significant others has been found to favour university students’ social interactions, while the family plays a critical role in improving psychological health (30).

A previous study suggested that students were less likely to be depressed or stressed with greater support from both family and friends (31). Besides, according to research, isolation and loneliness are reduced by having multiple sources of social support, especially among students (10). Perceived support from family and friends is higher than others as people may have more time to stay with their family members or friends living with or near them since people were advised to stay at home during the COVID-19 pandemic (32). This study supports the research findings that social support from family or friends are potential resources that can reduce the impact of an individual’s psychological problems, leading to psychological well-being.

In this study, it was found that the students’ perceived stress had a negative association with their perceived social support. The findings were supported by Bukhari and Afzal (13), and Ramezankhani et al. (14), which indicated a negative and significant correlation between perceived stress and perceived social support (*r* = −0.33, *P* < 0.001). Also, the finding is consistent with previous research studies that proved a low significant negative relationship between social support and level of stress (*r* = −0.43, *P* < 0.01), while university students with lower stress levels reported having higher social support (33).

Therefore, it is possible to argue that social support plays a role in dealing with stress. In this study, only 3.1% of undergraduate Health Sciences students reported low social support on the MSPSS. This is because social support may assist students in dealing with various pressures in their academic lives and enable a positive adjustment process in the COVID-19 circumstance. Thus, having a social support network would allow students to feel more capable of handling stress when it occurs. The findings of this study emphasise the vital role of social support received from family members in reducing the stress level among undergraduate Health Sciences students.

There are a few limitations to this study. Firstly, the study’s outcomes were constrained by the small number of students that participated from the Faculty of Health Sciences. This limits the applicability of the findings to other universities or faculties. Secondly, the lack of baseline data on undergraduate Health Sciences students’ mental health has become a limitation of this study. Thirdly, the study design used is a cross-sectional study that determines the association in this study. Thus, causality cannot be established. Finally, both PSS and MSPSS questionnaires were self-administered by the study participants. Hence, biases in self-reported data might be present. Future studies that involve other academic fields of study and qualitative research would give useful information on perceived social support among students.

## Conclusion

In conclusion, findings from this study revealed that social support from family, friends and significant others has an impact on students’ psychological well-being in which these supports were found to have a significant negative relationship with the stress level among students. The study also suggested that social support from family is the strongest for students to go through the stress of tough times. It also highlighted the need for attention to stress management among undergraduate students for healthy well-being.

## Figures and Tables

**Table 1:** Demographic characteristics of the participants (*N* = 290)

Variables	Frequency (*n*, %)
Gender	
Female	257 (88.6%)
Male	33 (11.4%)
Age	
19–21	58 (20%)
22–24	189 (65.2%)
25–28	43 (14.8%)
Year of study	
First-year	25 (8.6%)
Second-year	49 (16.9%)
Third-year	84 (29.0%)
Fourth-year	132 (45.5%)
Public universities	
UiTM	64 (22.1%)
USM	58 (20.0%)
UKM	58 (20.0%)
UniSZA	54 (18.6%)
UPM	56 (19.3%)

**Table 2:** The level of stress among undergraduate Health Sciences students (*N* = 290)

Level of stress	Frequency (*N* = 290)	Percentage %
High stress (27–40)	51	17.6
Moderate stress (14–26)	213	73.4
Low stress (0–13)	26	9.0

**Table 3:** Means and standard deviation of level of stress and perceived social support (*N* = 290)

Measures	Item	Mean	Standard deviation (SD)
PSS-10	Total score	21.17	5.75
MSPSS	Significant other	5.15	1.65
	Family	5.21	1.48
	Friends	5.34	1.33
	Total scale	5.23	1.19

**Table 4:** The correlation between perceived social support in MSPSS and level of stress in PSS-10 (*N* = 290)

	Total score MSPSS	Significant other	Family	Friend
Level of stress	−0.432**	−0.328**	−0.429**	−0.219**

Note:

** Correlation is significant at the 0.01 level (2-tailed)

**Table 5:** The correlation between perceived social support in MSPSS and level of stress in PSS-10 for different universities (*N* = 290)

Universities (level of stress)	Total score MSPSS
UiTM	−0.412**
USM	−0.401**
UKM	−0.411**
UniSZa	−0.409**
UPM	−0.370**
